# Neural Correlates of Theory of Mind in Autism Spectrum Disorder, Attention-Deficit/Hyperactivity Disorder, and the Comorbid Condition

**DOI:** 10.3389/fpsyt.2020.544482

**Published:** 2020-11-06

**Authors:** Daniel Ilzarbe, Steve Lukito, Carolin Moessnang, Owen G. O'Daly, David J. Lythgoe, Clodagh M. Murphy, Karen Ashwood, Vladimira Stoencheva, Katya Rubia, Emily Simonoff

**Affiliations:** ^1^Department of Child and Adolescent Psychiatry, King's College London (KCL), Institute of Psychiatry, Psychology and Neuroscience (IoPPN), London, United Kingdom; ^2^Department of Child and Adolescent Psychiatry and Psychology, Hospital Clínic de Barcelona, Institute of Neuroscience, Barcelona, Spain; ^3^Child and Adolescent Psychiatry and Psychology, August Pi i Sunyer Biomedical Research Institute (IDIBAPS), Barcelona, Spain; ^4^Department of Medicine, Universitat de Barcelona, Barcelona, Spain; ^5^Department of Psychiatry and Psychotherapy, Medical Faculty Mannheim, Central Institute of Mental Health, Heidelberg University, Mannheim, Germany; ^6^Centre for Neuroimaging Sciences, King's College London, London, United Kingdom; ^7^Behavioural and Developmental Psychiatry Clinical Academic Group, Behavioural Genetics Clinic, Adult Autism and Attention Deficit Hyperactivity Disorder Service, South London and Maudsley NHS Foundation Trust, London, United Kingdom; ^8^Department of Forensic and Neurodevelopmental Sciences, Institute of Psychiatry, Psychology and Neuroscience, King's College London, London, United Kingdom

**Keywords:** theory of mind (ToM), neurodevelopmental disorder, attention deficit and hyperactivity disorder (ADHD), autism spectrum disoder (ASD), functional magnetic resonance imaging (fMRI)

## Abstract

Theory of mind (ToM) or mentalizing difficulties is reported in attention-deficit/hyperactivity disorder (ADHD) and autism spectrum disorder (ASD), but the mechanism underpinning these apparently shared deficits is relatively unknown. Eighty-three young adult males, 19 with ASD alone, 21 with ADHD alone, 18 with dual diagnosis of ASD and ADHD, and 25 typically developing (TD) controls completed the functional magnetic resonance imaging version of the Frith-Happé animated-triangle ToM task. We compared neural function during ToM with two non-ToM conditions, random and goal directed motions, using whole-brain and region-of-interest analysis of brain activation and functional connectivity analyses. The groups showed comparable ToM task performance. All three clinical groups lacked local connectivity increase shown by TD controls during ToM in the right temporoparietal cortex, a key mentalizing region, with a differentially *increased* activation pattern in both ASD and comorbid groups relative to ADHD. Both ASD groups also showed reduced connectivity between right inferior lateral prefrontal and posterior cingulate cortices that could reflect an atypical information transmission to the mentalizing network. In contrast, with mentalizing both ADHD groups showed decreasing connectivity between the medial prefrontal and left temporoparietal cortices when compared to TD controls. Therefore, despite the complex pattern of atypical brain function underpinning ToM across the three disorders, some neurofunctional abnormalities during ToM are associated with ASD and appeared differentiable from those associated with ADHD, with the comorbid group displaying combined abnormalities found in each condition.

## Introduction

Autism spectrum disorder (ASD) is characterized by social communication difficulties and stereotypical/repetitive behaviors, while attention-deficit/hyperactivity disorder (ADHD) is characterized by age-inappropriate symptoms of inattention, hyperactivity, and impulsivity (1). Despite the distinct presentations, up to 55% of children and adolescents with the disorders meet both diagnostic criteria (2, 3). Furthermore, reports of a co-occurrence of both disorders in adulthood may indicate its persistence (4–6). Reviews and meta-analyses suggest overlapping cognitive impairments in both disorders including the difficulties in theory of mind (ToM), i.e., the inference of others' mental states or “mentalizing” (7–9), even though ToM deficits have been thought to be ASD-specific [e.g., (10–13)].

In ASD, mentalizing problems are conceptualized as a core symptom (11) or a consequence of diminished social motivation (14). The deficits in ASD are commonly associated with underactivation of temporo- and frontolimbic social brain networks, in medial and ventrolateral prefrontal, temporo-parietal cortices, and amygdala in children [e.g., (15, 16)] and adults [e.g., (17–19)], leading to the “hypo-intentionality” hypothesis of autism (20). However, overactivation of these regions has also been reported in children and adults with ASD (21–24). Reduced activation in the right temporoparietal junction (TPJ) and medial prefrontal cortex (mPFC), particularly, has been shown to correlate with increased autism severity scores (15, 17, 25).

During mentalizing, children and adults with ASD also showed reduced functional connectivity (FC) (17, 20, 21, 26, 27) in the social brain network that is typically increased in TD controls (17, 28, 29). Such FC reduction in ASD has been reported between frontal, especially mPFC, and temporo-parietal regions, particularly on the right hemisphere (20, 26, 30), ventral premotor and sensorimotor areas (25), and among widespread seed regions in the network (21, 27). These findings, together with observation of reduced FC across other cognitive domains (31–33), have led to the theory of domain-general frontal-posterior underconnectivity in ASD (34).

In ADHD, social cognition, and mentalizing deficits are increasingly reported, mostly in children (35–39) but also in adults (40–43), albeit not as consistently as in ASD (8). For the adult age group particularly, recent studies have reported intact mentalizing in ADHD, who are instead impaired in empathy and in their ability for generating solutions for social problems (44, 45). In line with these findings, a review has suggested that social cognition deficits in ADHD are less severe and more heterogeneous than in ASD (8).

In the context of neuroimaging of ADHD, altered orbitofrontal and lateral fronto-striatal activation, which is typicaly linked to executive function deficits (46–48), has been cited as possible mediator for the social cognition deficits in ADHD (49, 50), although, to our knowledge, no fMRI studies have explored neural correlates of ToM in ADHD. Importantly, it is unclear if social cognition and mentalizing deficits are intrinsic to ADHD (41) or reflect co-occuring ASD traits in the population (40).

Comparative fMRI studies involving people with ADHD alone, ASD alone, and/or combined ASD+ADHD could tease apart disorder-differentiating impairments in social brain regions. To date, these studies have been conducted exclusively in children and adolescents with the disorders (51–55). One functional magnetic resonance imaging (fMRI) study has found ASD-specific inferior frontal gyrus underactivation relative to TD and ADHD during emotion identification (51). Furthermore, using resting-state fMRI, ASD-differentiating intrinsic FC impairments were observed in regions typically involved in mentalizing, with increased *local* (i.e., “degree centrality”) temporo-limbic FC in the ASD and ASD+ADHD groups relative to TD and ADHD, and shared precuneus overconnectivity across all clinical groups (55). Electroencephalography (EEG) biomarkers for face and eye-gaze processings such as N170 have also been found to be ASD-differentiating relative to ADHD (52–54). These findings suggest that the neural abnormalities underlying social cognition, based on a number of neural and electrophysiological correlates, might be specific to ASD and not ADHD. However, the hypothesis has not been tested during mentalizing specifically.

Therefore, we directly compare hemodynamic response and FC in brain regions during ToM relative to non-ToM conditions across individuals with ADHD, ASD, ASD+ADHD, and typical development. To address the lack of investigation of brain function correlates of comorbidity in the adult population, this study focused on young adults using the Frith-Happé animated-triangle fMRI task that is frequently used for investigating ToM in ASD (21, 24, 27), which has also been shown to detect ToM deficits in ADHD, albeit in children (35). We aimed to investigate if ToM performance deficits are similar or different and are associated with similar or different neural correlates across the three clinical groups. Based on previous findings of neuroimaging (51), FC (55), and EEG (52–54), we hypothesized that abnormalities in the social brain network, e.g., under-activation in right TPJ and mPFC, and reduced fronto-posterior FC during ToM (21, 27, 34) would distinguish the ASD groups, with and without ADHD, from the group with ADHD alone, who would show no neurofunctional impairments.

## Materials and Methods

### Participants

A total of 107 young adult males aged 20–27 years with ASD, ADHD, ASD+ADHD, and TD and full-scale intelligent quotient (FSIQ) ≥70, estimated using the Wechsler Abbreviated Scale of Intelligence (56), took part in the study. Only males were included to increase group homogeneity for these predominantly male-prevalent disorders (57, 58). Handedness was assessed using the Edinburgh Handedness Inventory (59) and did not differ between groups. Excluded were participants with epilepsy, personality disorder, current substance abuse/dependence, lifetime history of bipolar disorder, schizophrenia or head injury, or contraindication for fMRI. The participants completed a larger study involving several fMRI tasks and a neurocognitive task battery (60). After excluding 24 subjects (four with incomplete fMRI data, seven with missing performance data due to technical or compliance issues [these participants answered ≤3 out of 12 questions], 12 with excessive movement (>3 mm) and one with an incidental MRI finding), 83 subjects remained (19 ASD; 21 ADHD; 18 ASD+ADHD; 25 TD). The groups differed in FSIQ, but not age (See Table 1 for descriptive statistics).

**Table 1:** Group differences in socio-demographic variables and clinical measures.

	**TD** (***n*** **=** **25)**		**ASD** (***n*** **=** **19)**		**ASD+ADHD** (***n*** **=** **18)**		**ADHD** (***n*** **=21)**		**Group** **comparison**			***Post-hoc***
	**M**	**SD**	**M**	**SD**	**M**	**SD**	**M**	**SD**	***F/t***	***df***	***p***	
Age	23.4	1.6	23.0	0.7	23.1	1.3	23.1	2.1	0.22	3, 79	0.88	–
FSIQ	116.8	11.9	105.2	18.9	106.9	14.0	117.8	13.1	4.2	3, 79	0.009	ADHD^*^, TD^*^ > ASD
Handedness	64.8	70.4	64.7	69.9	71.1	59.0	59.5	70.9	0.09	3, 79	0.96	–
**CAARS ADHD index (*****t*****-scores)**												
Self-rated	45.0	7.3	49.4	8.0	58.2	11.3	66.2	6.7	27.9	3, 78	<0.001	ADHD^***^, ASD+ADHD^*^ > ASD; ADHD^***^, ASD+ADHD^***^ > TD; ADHD^*^ > ASD+ADHD
Informant-rated	–	–	46.7	5.1	66.1	11.3	62.5	11.0	21.9	2, 53	<0.001	ADHD^***^, ASD+ADHD^***^ > ASD
**SDQ hyperactivity/inattention (raw scores)**												
Self-rated	1.8	1.4	3.1	2.2	6.6	2.2	7.3	1.6	47.6	3, 78	<0.001	ADHD^***^, ASD+ADHD^***^ > ASD; ADHD^***^, ASD+ADHD^***^ > TD; ASD^†^ > TD
Informant-rated	–	–	2.8	1.5	7.2	1.7	7.6	1.7	48.8	2, 52	<0.001	ADHD^***^, ASD+ADHD^***^ > ASD
**ADHD symptom counts**^**(a)**^												
Inattention	–	–	–	–	7.4	1.2	8.1	1.5	−1.5	1, 32	0.15	–
Hyperactivity/impulsivity	–	–	–	–	4.8	2.7	4.7	2.4	0.03	1, 32	0.98	–
**Total SRS-2 (*****t*****-scores)**												
Self-rated	48.2	5.9	61.1	9.7	63.7	10.6	61.5	7.4	16.3	3, 78	<0.001	ASD^***^, ASD+ADHD^***^, ADHD^***^ > TD
Informant-rated	–	–	62.8	6.0	69.5	12.9	58.0	11.3	5.6	2, 53	0.006	ASD+ADHD^**^ > ADHD
**ADOS-2 Module 4**^**(b)**^												
Communication	–	–	1.7	1.9	2.4	2.4	–	–	−1.0	1, 30	0.33	–
Social interaction	–	–	3.3	2.7	4.0	4.2	–	–	−0.60	1, 30	0.56	–
Communication + social interaction	–	–	4.9	4.0	6.4	6.5	–	–	−0.79	1, 30	0.43	–
Stereotyped behaviors and restricted interest	–	–	0.3	.96	1.0	1.4	–	–	−1.7	1, 30	0.10	–

TD, typical development; ASD, autism spectrum disorder; ADHD, attention-deficit/hyperactivity disorder; M, mean; SD, standard deviation; df, degree of freedom; FSIQ, full-scale intelligence quotient; CAARS, Conners Adult ADHD Rating Scale; SRS, Social Responsiveness Scale version 2; SDQ, Strengths and Difficulties Questionnaires, a version for adults was used. ^(a)^Current ADHD symptom counts were based on the Diagnostic Interview for Adult ADHD (DIVA 2.0) or the Young Adult Psychiatric Assessment, available in 18 participants with ADHD and 16 participants with ASD+ADHD. ^(b)^Current Autism Diagnostic Observation Schedule (ADOS-2) scores were available in a subset of 18 individuals with ASD and 14 participants with ASD+ADHD. Post-hoc significant threshold:

* *p < 0.05*,

** *p < 0.01*,

*** p < 0.001, and

† *p < 0.1 with Tukey–Kramer multiple comparison correction*.

The clinical groups were recruited through specialist adult ASD and ADHD clinics, support organizations, social media, and from a well-characterized population-based cohort of young adults with ASD, the Special Needs and Autism Project (SNAP), who were followed up since 10–12 years and are now young adults (61). Psychostimulant medications, which were withdrawn 48 h before testing, and selective serotonin reuptake inhibitors (SSRIs) prescription, were not exclusion criteria for the clinical groups. Participants with non-stimulant ADHD medications were excluded due to their relatively longer withdrawal periods.

The ASD group consisted of 14 participants with clinical diagnoses (six autism, eight Asperger's syndrome) and five participants with research diagnoses from SNAP [two autism, two atypical autism, and one pervasive developmental disorder (PDD) unspecified], based on the International Classification of Diseases criteria (ICD-10) (62). All but one diagnoses were accompanied by gold-standard research instruments, the Autism Diagnostic Observation Schedule (ADOS) (63) or parent interviews on the Autism Diagnostic Interview-Revised (ADI-R) (64). Where available, current ADOS scores were collected from the examiners. One participant received childhood ASD diagnosis from a specialist neurodevelopmental clinic, supported by an assessment on the Diagnostic Interview for Social and Communication Disorders (DISCO) (65), but had no current scores. None were prescribed medication.

In the ASD+ADHD group, 14 subjects had clinical diagnoses (five autism, six Asperger's syndrome, three atypical autism) and four had research diagnoses of ASD from SNAP (two atypical autism, two PDD unspecified) based on the ICD-10. All but one ASD diagnoses were accompanied by the ADOS or the ADI-R (ADOS score on Table 1). One participant's childhood ASD diagnosis from a specialist neurodevelopmental clinic was supported by the DISCO (no current scores). Furthermore, thirteen participants met the criteria for combined and five for inattentive DSM-5 ADHD subtype. Nine had current clinical DSM-5 ADHD diagnoses supported by current assessments on the Diagnostic Interview for Adult ADHD (DIVA 2.0) (66) or the Conners' Adult ADHD Diagnostic Interview for DSM-IV (CAADID) (67). Nine other participants had a significant history of ADHD symptoms assessed through SNAP and met the current ADHD DSM-5 criteria on the Young Adult Psychiatric Assessment (68). Two were prescribed psychostimulants alone [methylphenidate (MPH), dexamphetamine], one SSRIs alone (escitalopram) and one both (MPH, sertraline).

All participants in the ADHD group met the DSM-5 diagnostic criteria: 12 with combined, eight inattentive, and one hyperactive subtype, diagnosed by consultant psychiatrists in specialist adult ADHD clinics. Eighteen diagnoses were supported by the DIVA 2.0 and three by the CAADID. Four were prescribed psychostimulants alone (MPH and lisdexamphetamine), one with SSRIs alone (sertraline) and one with both (MPH and sertraline).

The TD participants were from local communities, were non-medicated, and scored below clinical cut-off on the Conners' Adult ADHD Rating Scale (CAARS) (69) and the Social Responsiveness Scale-2 (SRS-2) (70) for ADHD and ASD traits, respectively. This study was in accordance of the Declaration of Helsinki and had ethical approval from a local National Health Service Research Ethics Committee (NHS REC 13/LO/0373). Each participant gave written informed consent and was given £50 and travel reimbursement.

### Clinical Measures

The CAARS ADHD index and the hyperactivity/inattention domain of the Strengths and Difficulties Questionnaires (SDQ) for adults (http://www.sdqinfo.com) indexed ADHD traits, while total algorithm score of SRS-2 indexed ASD traits. The participants completed self-report measures, corroborated by informant (e.g., parents/partner/siblings) for those in the clinical groups.

### The Frith-Happé Animated Triangles Task

The block-design fMRI version of the Frith-Happé task was selected as it evokes large effect size of brain activation, which is associated with increased statistical power. The task was also selected based on its frequent use in ToM investigation in ASD children and adult populations (21, 24, 27). A recent study furthermore reported ToM deficits in ADHD children using the same task (35). The task consists of twelve 26–48 s cartoons involving two triangles whose movements express: (1) ToM, e.g., persuading (length = 39.0±2.2s), (2) Goal-directed (GD) interaction, e.g., following (length = 39.5 ± 9.5 s), and (3) Random (RD) purposeless motions, e.g., floating (length = 39.8 ± 1.5 s) (71). Each condition is depicted by four different clips, shown in the same pseudo-randomized block order across participants. Each block consists of 1-s fixation cross (jittered between 0.3–1.9 s), one clip, a 3-s fixation and a visual multi-choice question prompting the participants to identify the movement type shown with a maximum duration of 5 s. The chosen answer is highlighted for 1 s (Supplementary Figure 1) before the start of the next block. Response accuracy and response time (RT) were collected during the task.

Outside the fMRI scanning, a subset of the clips (four ToM and four GD) was shown to the participants, followed by the question “*What do you think is happening during the clip?”* Neutral prompts (e.g. “uh-hum”) were given by the examiner when the answer was not forthcoming. Responses were transcribed and rated by the first author DI, blinded to the participants' diagnoses, yielding scores for intention attributions (0–5), their appropriateness (0–3), the primary measures; and response length (i.e., number of clauses) and number of prompts, our secondary measure of ToM ability (72). Forty-eight randomly selected transcriptions (12 per group) were independently rated by the second author SL, achieving good interrater agreement for ratings of intention attribution (weighted κ =.71; weighted agreement = 92.8%), appropriateness (weighted κ =.69; weighted agreement = 88.6%), response length [intra-class correlation coefficient (ICC) = 0.97], and number of prompts (ICC = 0.99). Behavioral data were analyzed in STATA 14.0 (73) using (Group × Condition) repeated measures ANOVA, with *post-hoc* analyses corrected using the Tukey–Kramer method, accounting for unequal sample sizes across groups.

### Neuroimaging Data Acquisition

Data were acquired using a General Electric (GE) MR750 3T scanner (General Electric, Boston, MA, United States) at the Center for Neuroimaging Sciences, King's College London. The scanner's body coil was used for RF transmission, while an eight-channel head coil was used for signal reception. An echo planar image (EPI) gradient-echo pulse sequence (TR/TE = 2,000/30 ms, flip angle = 80°, FOV = 19.2 × 19.2 cm, 64 × 64 matrix, in-plane resolution = 3 mm, slice thickness/gap = 3/0.3 mm) was used to acquire 40 slices of T2^*^-weighted MR images angled at 20° up from inter-commissural plane, prescribed consecutively top-to-bottom, covering the entire brain. The 10-min, 14-s task produced 307 volumes in time series. A whole-brain high resolution structural T1-weighted scan (Sagittal ADNI-GO/2 ACC MPRAGE), co-registered with individual activation maps during pre-processing, was acquired in the inter-commissural plane with TE = 3.016 s, TR = 7.312 s, 196 slices, FOV = 27 cm × 27 cm, 256 × 256 matrix, and slice thickness of 1.2 mm.

### Neuroimaging Analyses

fMRI data were corrected for slice timing, realigned, co-registered to the individual T1-weighted scan, segmented, normalized to the Montreal Neurological Institute EPI template, and smoothed using an 8-mm Gaussian kernel. Statistical analyses were completed in two steps on the Statistical Parametric Mapping (SPM8). At the subject-level analyses, BOLD response was predicted using a vector of onsets and durations convolved with the canonical hemodynamic response function (HRF). Six nuisance motion regressors [x-, y-, z-translations, rotations, and additional regressors for each motion spike (>1 mm)] controlled for volume-to-volume head motion and abrupt movements. A high-pass filter was applied at the cut-off of 128 s and a first-order autoregressive model corrected for time series correlation. Investigations were carried out in the orthogonal contrast ToM > RD, typical of past studies (21, 27, 74–76), and separately in ToM > GD, a higher-level contrast investigated in a recent study (72).

Both contrasts were entered to second-level analyses. Within-group activations were analyzed with a cluster extent threshold of *p* <.05, family-wise error corrected (FWE_cor_), and a cluster-forming voxel threshold of *p* < 0.001 (**Figure 2**). Between-group activation was modeled with group as predictor, covarying for total frame-wise head displacement to control for residual motion variation. The group differences were analyzed, first, using an exploratory whole-brain analysis (voxel threshold *p* < 0.05, FWE_cor_) and, second, using a hypothesis-driven region-of-interest (ROI) analyses, by means of small-volume correction, using 10-mm radius spherical ROIs in the bilateral inferior frontal gyrus (IFG), bilateral medial prefrontal cortex (mPFC), bilateral posterior cingulate cortex (PCC), bilateral angular gyrus (ANG), and bilateral temporo-parietal junction/superior-temporal sulcus (TPJ/STS). The ROIs were centered on independently derived coordinates from a whole-brain activation map of ToM > RD [http://neurovault.org/images/3180 (77)], following Kana et al. (27). Mean beta weights of BOLD data were extracted for *post-hoc* pairwise group comparisons, applying Tukey–Kramer correction, and for correlational analyses with primary trait and task performance measures, applying false-discovery rate [FDR] correction. Logarithmic transformations were applied to normalize skewed data distribution as appropriate.

To compare FC between task conditions, we investigated the synchronization of BOLD activation time series across regions to facilitate comparison with previous studies [e.g., (21, 26, 27, 78)]. Activation time-series were first extracted from the spherical ROIs for everyone. The activation time series in ipsilateral TPJ and STS were combined by averaging due to regional overlap, yielding eight seed regions for the subsequent FC analyses. To control for artifacts, six orthogonal head motion parameters, head movement spikes, white matter, and cerebrospinal fluid signal plus their derivatives were regressed out. Artifact-corrected time-series were segmented pertaining to each video clip and those representing the same movement condition were concatenated into a condition-specific time series (27). These time series were correlated pairwise between ROIs, yielding altogether 28 unique correlation coefficients per movement condition (i.e., ToM, GD, and RD), which were then Fisher's *z*-transformed using an inverse hyperbolic tangent function to produce a FC strength index. They were then analyzed using multilevel mixed-effects linear regression models with group, condition, and group × condition as fixed effects and individual factor as random effects. Model fit was first examined for the overall averaged FC, calculated by averaging *z*-scores from the 28 pairwise correlations between ROIs. Further examination of model fit then took place in each pairwise FC. Group × condition interaction effects and main effects of group and condition were explored in models with significant fixed-effect model fit. Then, significant *post-hoc* simple effect testing was performed and corrected for multiple testing using the FDR method. Analyses were repeated, covarying for FSIQ and medications in each model to assess their influence in each model. Finally, Pearson's correlations assessed the relation between FC strength, primary measures of clinical traits, and task performance during ToM, corrected for multiple testing with the FDR method.

## Results

### Task Performance Results

**Figure 1:**
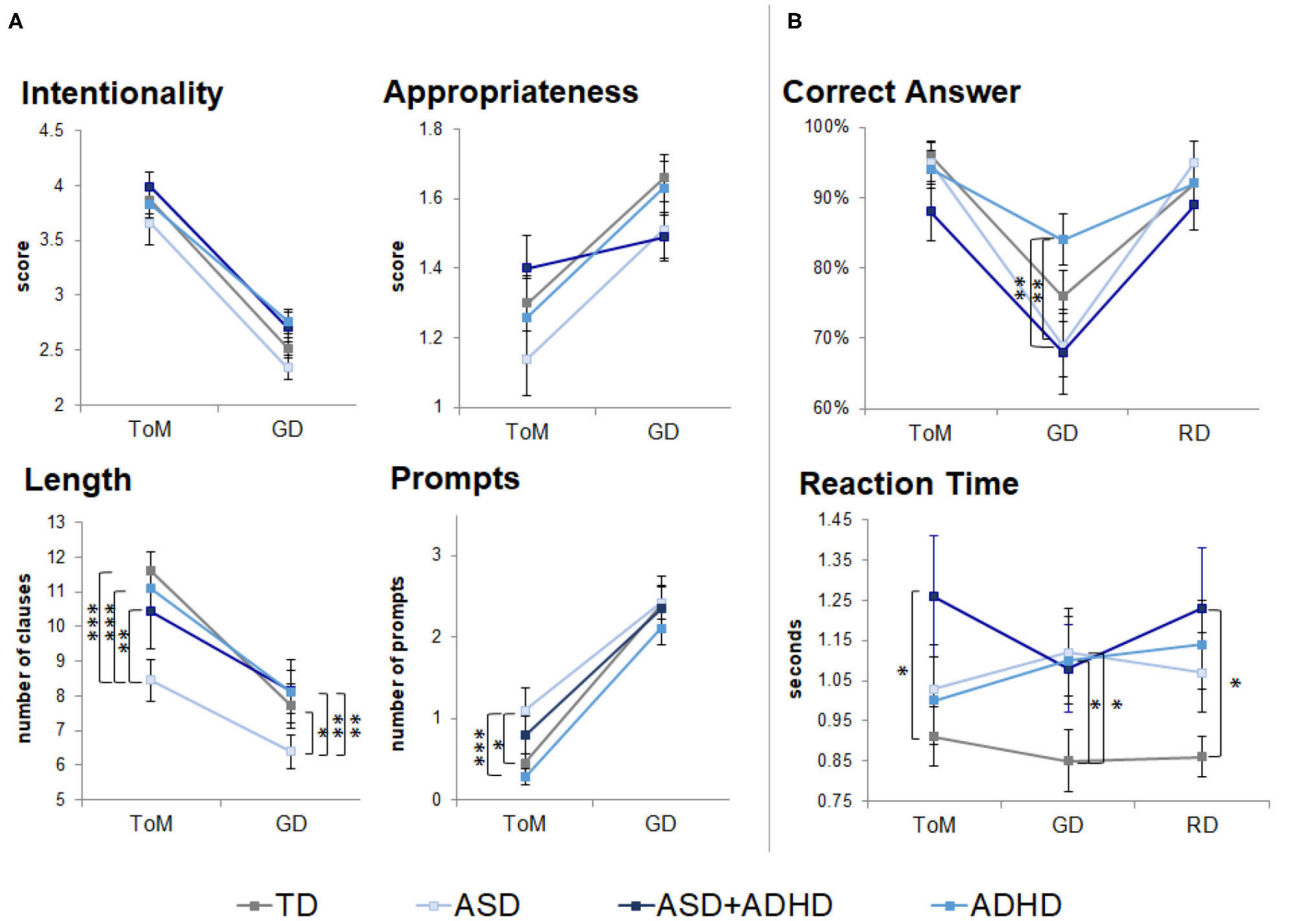
Behavioral performance outside (neuropsychological task) and during fMRI scanning (fMRI task). TD, typical development; ASD, autism spectrum disorder; ADHD, attention-deficit/hyperactivity disorder; GD, goal directed; RD, random movement; ToM, theory of mind. Only pairwise group differences are highlighted although an effect of condition was found in scores for intentionality, appropriateness, length of descriptions and number of prompts during neuropsychological task. An effect of condition was also found for correct identification of motion (correct answers) during the fMRI task. *p < 0.05, **p < 0.01, ***p < 0.001, with Tukey–Kramer multiple comparison correction. Error bars represent standard error of the mean. **(A)** Neuropsychological task behavioral results. **(B)** fMRI task behavioral results.

In the performance data from the out-of-scanner task, there were no significant group × condition interactions (Supplementary Table 1). No group differences were found in ratings of intention attributions [*F*(3, 77) = 2.09, *p* = 0.1] and their appropriateness [*F*(3, 77) = 0.88, *p* = 0.5]. A group effect was found on the length of description [*F*(3, 77) = 2.73, *p* < 0.05] and prompts required [*F*(3, 77) = 3.48, *p* = 0.02; Figure 1A, Supplementary Table 1]. *Post-hoc t*-tests showed that the ASD group gave the shortest ToM descriptions (*p*s < 0.01), despite receiving more prompts than the ADHD (*p* < 0.01) and TD groups (*p* < 0.05). An effect of condition was found on the intentionality scores [*F*(1, 77) = 239.0, *p* < 0.0001], appropriateness scores [*F*(1, 77) = 33.3, *p* < 0.0001], length of description [*F*(1, 77) = 117.3, *p* < 0.0001] and prompts [*F*(1, 77) = 184.3, *p* < 0.0001]. *Post-hoc t*-tests suggested that intentionality scores and length of description were higher in ToM than GD across all groups (*p* < 0.001); appropriateness scores were lower in ToM than GD in the TD, ASD, and ADHD groups (*p* < 0.001); and more prompts were needed during ToM than the GD condition (*p* < 0.0001).

During the fMRI task, a participant with ADHD answered eight out of 12 questions (others answered ≥11 questions), which could reflect ToM difficulties. The participant was included in all analysis. There were no significant group × condition interactions (Supplementary Table 1). A trend-level group effect [*F*(3, 79) = 2.49, *p* = 0.07] and a significant effect of condition [*F*(2, 158) = 42.3, *p* < 0.0001] were found for motion type identification (Figure 1B, Supplementary Table 1). *Post-hoc t-*tests showed GD motion were identified more accurately by the ADHD than the ASD and ASD+ADHD groups (*p* < 0.01), and that RD and ToM motions were identified more accurately than GD motions (*p* < 0.001) by the TD, ASD and ASD+ADHD groups. RT comparisons showed a trend effect of group [*F*(3, 79) = 2.60, *p* = 0.06], with the clinical groups being slower than the TD group, although this was only statistically significant in RD and ToM conditions for the ASD+ADHD group (*p* < 0.05) and in GD for the ASD and ADHD groups (*p* < 0.05) when tested *post-hoc*.

### Motion

Total volume-to-volume head movement in the x, y, and z rotation and translation did not differ across groups [*F*(3, 79) = 0.74, *p* = 0.53].

### Within-Group Brain Activation

**Figure 2:**
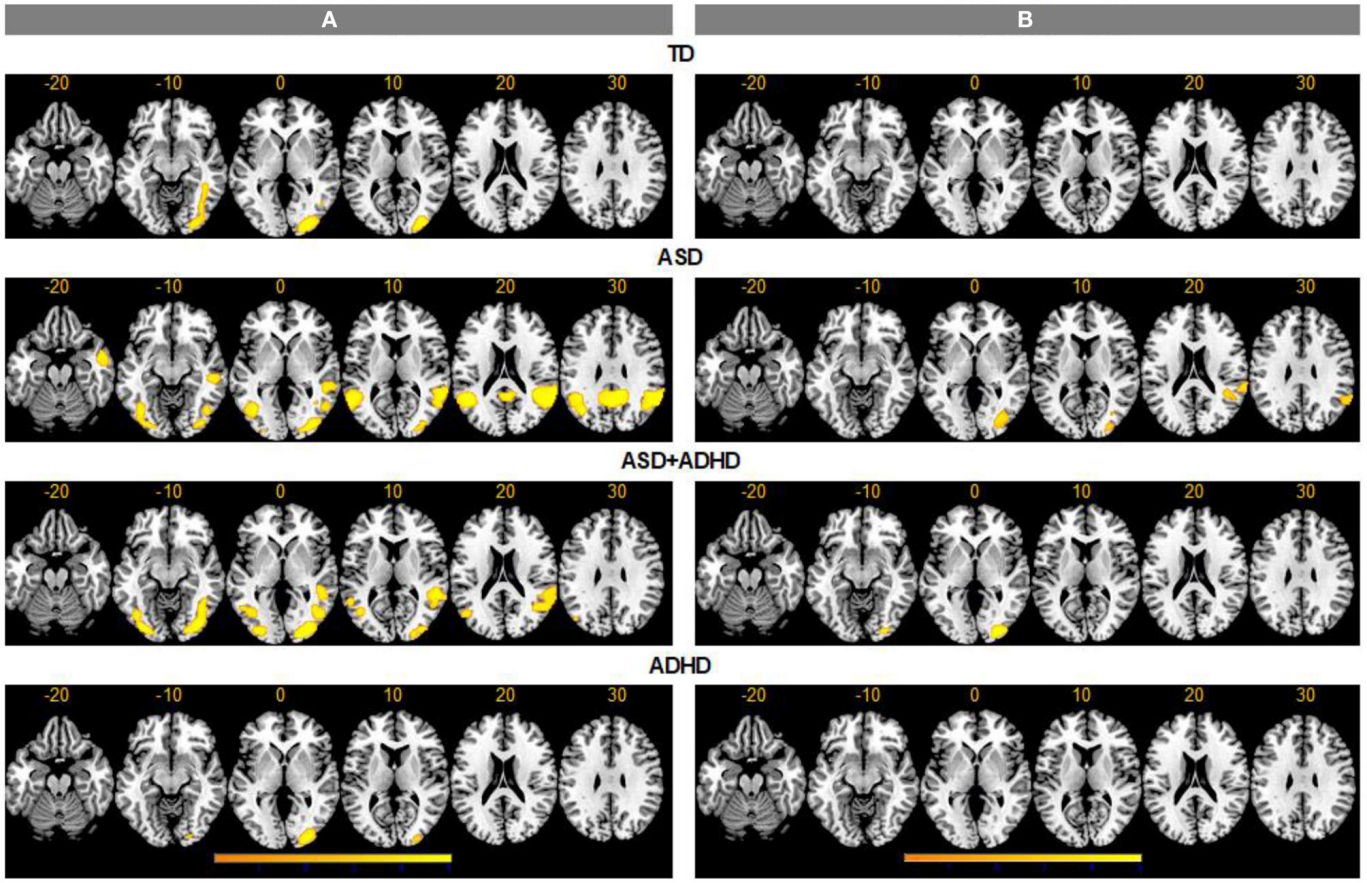
Activation clusters for theory of mind (ToM) over random (RD) and goal-directed (GD) motion experimental conditions. TD, typical development; ASD, autism spectrum disorder; ADHD, attention-deficit/hyperactivity disorder. **(A)** ToM > RD, **(B)** ToM > GD.

For the ToM > RD contrast, TD controls showed increased activation in ToM relative to RD condition in a region extending from inferior/middle occipital gyri (IOG/MOG) to posterior inferior temporal gyri (ITG) (BA19/37). In ASD, increased activation for ToM > RD was observed in bilateral IOG/MOG/ITG, in precuneus/posterior cingulate (BA7/23), and in bilateral supramarginal/ANG/posterior superior temporal gyrus (STG), extending to right middle temporal gyrus (MTG)/temporal pole (BA 22/21/38). In ADHD, increased activation for ToM > RD was found in right occipital pole, while in ASD+ADHD, the increased activation extended from bilateral IOG/MOG/ITG to right fusiform and lingual gyri, and from bilateral posterior MTG to right posterior STG/supramarginal gyrus/ANG (BA42/39/21/22) (Figure 2).

For the ToM > GD contrast, the TD and ADHD groups showed no clusters of increased activation at the chosen threshold. In ASD, increased activation was observed in right supramarginal/ANG/posterior STG (BA 42/41/40/22/48) and right IOG/MOG reaching into fusiform gyrus. In ASD+ADHD, increased activation was found in right IOG/MOG/lingual gyri (Figure 2).

### Between-Group Brain Activation

**Figure 3:**
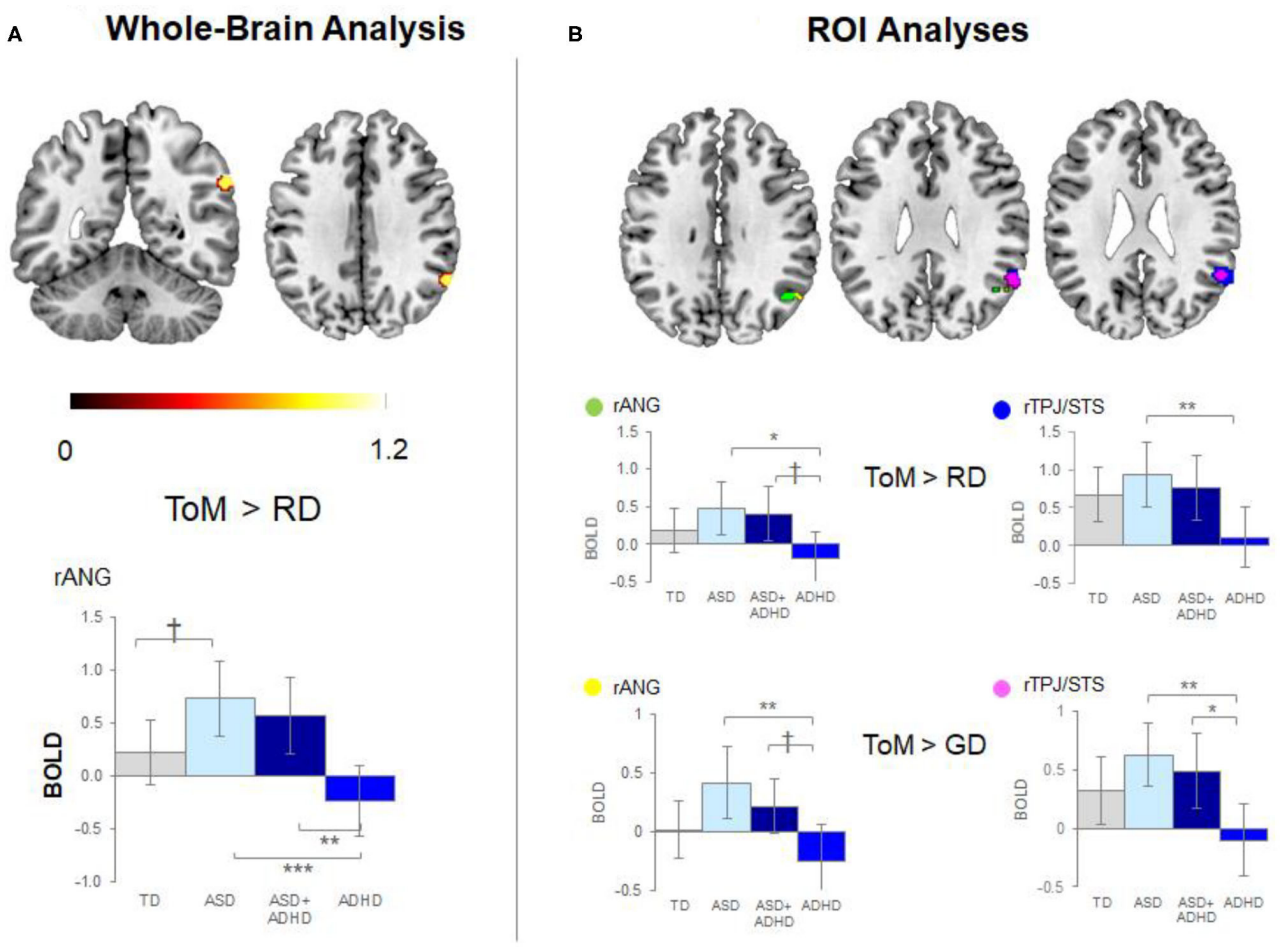
Significant peak activation by clusters and bar graphs showing within pairwise comparisons. **(A)** Whole-brain analysis and **(B)** Region of interest (ROI) analyses. TD, typical development; ASD, autism spectrum disorder; ADHD, attention-deficit/hyperactivity disorder; BOLD, blood-oxygen-level dependent; GD, goal directed; RD, random movement; ToM, theory of mind; rANG, right angular gyrus; rTPJ/STS, right temporoparietal junction/superior temporal sulcus *p < 0.05, **p < 0.01, ***p < 0.001, †*p* < 0.1 with Tukey–Kramer multiple comparison correction.

Whole-brain analysis revealed no group effect for the contrast of ToM > GD. A group effect was observed for ToM > RD in the rANG [*p* =.005, *F* = 12.5, (x = 60, y = −54, z = 34), *cluster size* = 40 voxels]. *Post-hoc* pairwise comparisons revealed lower right ANG activation in the ADHD than the ASD (*p* < 0.001) and ASD+ADHD (*p* = 0.009) groups, and a trend-level increase in ASD relative to TD (*p* = 0.058; Figure 3A, Table 2).

**Table 2:** Significant peak activation by cluster for the contrasts theory of mind (ToM) over random (RD) and goal-directed (GD) motion in the whole-brain and region-of-interest (ROI) analyses.

	**Voxels**	**MNI coordinates**			***F***	***Z*_**score**_**	***p*_**FWEcorr**_**	***Post-hoc***
		**x**	**y**	**Z**				
**Contrast ToM** **>** **RD**								
**Whole-brain analysis**								
rANG	40	60	−54	34	12.5	4.76	0.005	ASD^***^, ASD+ADHD^**^ > ADHD; ASD^†^ > TD
**ROI analyses**								
rTPJ/STS	78	58	−52	30	10.1	4.24	<0.001	ASD^**^ > ADHD
rANG	49	50	−60	34	5.58	2.95	0.026	ASD^*^, ASD+ADHD^†^ > ADHD
		44	−62	36	5.34	2.86		
**Contrast ToM** **>** **GD**								
**Whole-brain analysis**								
–								
**ROI analyses**								
rTPJ/STS	25	54	−54	28	5.49	2.91	0.069	ASD^**^, ASD+ADHD^*^ > ADHD
		58	−52	30	5.31	2.85		
rANG	4	52	−60	32	4.98	2.72	0.063	ASD^**^, ASD+ADHD^†^ > ADHD

TD, typical development; ASD, autism spectrum disorder; ADHD, attention deficit/hyperactivity disorder; ToM, theory of mind; RD, random movement; rANG, right angular gyrus; rTPJ/STS, right temporo-parietal function/superior temporal sulcus. MNI, Montreal Neurological Institute;

*** *p < 0.001*,

** *p < 0.01*,

* p < 0.05, and

† *p < 0.1, with Tukey–Kramer multiple comparison correction*.

Between-group ROI analyses of ToM > RD revealed a group effect in rTPJ/STS [*p* < 0.001, *F* = 10.1, (x = 58, y = −52, z = 30), k = 78 voxels] and rANG [*p* =.026, *F* = 5.6, (x = 50, y = −60, z = 34), k = 49 voxels]. *Post-hoc t*-tests revealed significant higher activation in ASD than ADHD (*p*s ≤ 0.016) in both regions and a trend-level increase in rANG in ASD+ADHD relative to ADHD (*p* = 0.070). Corresponding analyses of ToM > GD contrast showed a trend-level group effect in rTPJ/STS [*p* = 0.069, *F* = 5.5, (x = 54, y = −54, z = 28), k = 25 voxels] and rANG [*p* = 0.063, *F* = 5.0, (x = 52, y = −60, z = 32), k = 4 voxels], with higher activation in ASD than ADHD (*p*s ≤ 0.004) for both regions, and a higher increase in ASD+ADHD than ADHD in rTPJ/STS (*p* = 0.025) and a trend-level increase rANG (*p* = 0.067; Figure 3B, Table 2).

In all analyses, the effects of FSIQ (*p*s ≥ 0.34), prescription of SSRI alone (*p*s ≥ 0.38), psychostimulant alone (*p*s ≥ 0.49), and all medication together (*p*s ≥ 0.17) were non-significant.

In the TD group, RT to ToM clips showed significant positive correlation with ROI-based activation, for the ToM > RD and ToM > GD contrasts, respectively, in rANG (*r* = 0.59, *p* = 0.008, *r* = 0.46, *p* = 0.021) and rTPJ/STS (*r* = 0.55, *p* = 0.014, *r* = 0.52, *p* = 0.016) and in the whole-brain analysis based rANG cluster for ToM > RD (*r* = 0.66, *p* = 0.002). In the ASD group, the number of prompts during ToM was significantly positively correlated with activation in rANG (*r* = 0.58, *p* = 0.022), and at trend-level, in the whole-brain rANG cluster (*r* = 0.44, *p* = 0.071) in the ToM > RD contrast; and with ROI activation in rANG (*r* = 0.63, *p* = 0.020) and, at a trend-level, in rTPJ (*r* = 0.45, *p* = 0.093) for the ToM > GD contrast.

None of the activation clusters correlated with the severity of ASD or ADHD traits (*ps* ≥ 0.26).

### Functional Connectivity

Analyses of overall averaged FC revealed significant effects of condition [χ^2^(2) = 7.79, *p* = 0.02] and of group × condition interaction [χ^2^ (6) = 16.3, *p* = 0.012] but not group. *Post-hoc t*-test showed increased overall FC during ToM relative to RD or GD in TD controls only (*p*s ≤ 0.001), although the pattern of findings subtly differed for the individual FC pairs.

**Figure 4:**
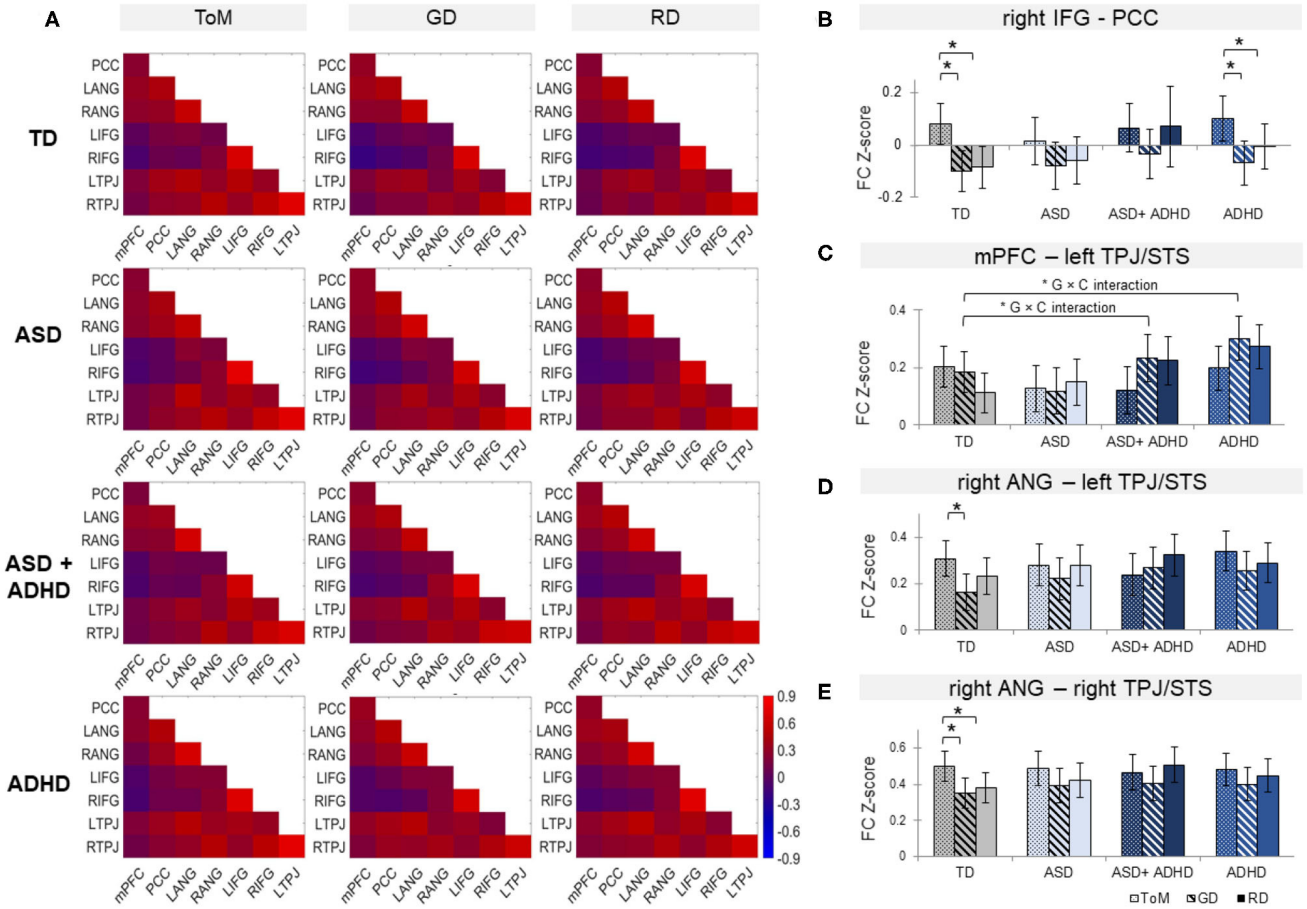
**(A)** Functional connectivity (FC) matrices within each group for theory of mind (ToM), goal-directed (GD), and random (RD) movement among medial Prefrontal Cortex (mPFC), posterior cingulate cortex (PCC), bilateral angular gyrus (ANG), bilateral inferior frontal gyrus (IFG), and bilateral temporoparietal junction (TPJ), combined with superior temporal sulcus (STS). Bar graphs of FC for right IFG-PCC **(B)**, mPFC-left TPJ/STS **(C)**, right ANG with left **(D)**, and right TPJ/STS **(E)** by group and condition. *G × C represents significant group by condition interaction. Error bars represent 95% confidence interval. TD, typically developing controls; ASD, autism spectrum disorder; ADHD, attention-deficit/hyperactivity disorder. *p < 0.05 with false-discovery rate multiple comparison correction.

Among the ROI pairs, a significant mixed-effect model fit was found between rIFG and PCC, [χ^2^(11) = 46.6, *p* < 0.0001); and trend-level significance between mPFC and lTPJ [χ^2^(11) = 27.7, *p* = 0.051]; and between rANG with lTPJ [χ^2^(11) = 24.9, *p* = 0.076] and rTPJ [χ^2^(11) = 24.5, *p* = 0.076] (see Figure 4). Between rIFG and PCC, there was a significant effect of condition [χ^2^(2) = 36.4, *p* < 0.0001], qualified by increased FC during ToM relative to RD or GD in the TD (*p*s ≤ 0.0004) and the ADHD groups (*p*s ≤ 0.04). Between mPFC and lTPJ, a significant group × condition interaction [χ^2^(6) = 14.5, *p* = 0.024) and a significant group effect [χ^2^(3) = 9.3, *p* = 0.025) were found. *Post-hoc* analyses for the interaction showed FC increase in TD controls and decrease in ASD+ADHD and ADHD (*p*s ≤ 0.007) during ToM relative to RD; and comparable FC during ToM and GD in TD controls, and FC decrease during ToM relative to GD (*p*s ≤ 0.045) in ASD+ADHD and ADHD. *Post-hoc* analyses for the group effect showed increased FC in ADHD relative to TD controls during RD condition (*p* = 0.024), and relative to ASD during the GD condition (*p* = 0.024). Between rANG and bilateral TPJ, there was a significant effect of condition [rANG-lTPJ: χ^2^(2) = 10.2, *p* = 0.006; rANG-rTPJ χ^2^(2) = 15.8, *p* = 0.0004] due to increased FC in TD controls during ToM relative to RD or GD (*p*s ≤ 0.025) between rANG and rTPJ; and during ToM relative to GD (*p* = 0.002) between rANG and lTPJ. No other effects or interactions were significant.

The FSIQ (*p*s ≥ 0.10), SSRIs alone (*p*s ≥ 0.72), psychostimulants alone (*p*s ≥ 0.42), and all medications together (*p*s ≥ 0.38) exerted non-significant effects in the models.

The FC strength neither correlated with the severity of ASD and ADHD traits (*p*s >0.39) nor task performance measures (*p*s > 0.11).

## Discussion

To our knowledge, this is the first study to investigate brain activation and FC associated with ToM in young adult males with ASD, ADHD, ASD+ADHD, and TD. Despite comparable task performance, ASD adults with and without ADHD had increased activation relative to ADHD alone during mentalizing in a key temporo-parietal ToM region, which was trend-wise increased relative to TD in the ASD group without ADHD. In FC, during mentalizing, there were mixed patterns of findings with all clinical groups sharing the lack of increased average FC over all connectivity pairs when compared to TD, which was particularly significant when considering the individual FC pairs in posterior temporo-parietal regions. Furthermore, underconnectivity between right inferior frontal and posterior cingulate cortices was found in the ASD groups, with and without ADHD, while the decreasing FC in medial frontal cortex and left temporo-parietal junction with mentalizing was found only in the ADHD groups, with and without ASD.

Increased temporoparietal activation in rANG during mentalizing appears to differentiate adults with ASD, with or without ADHD, from those with ADHD alone, and at a trend-level from TD controls. Therefore, we show for the first time that this overactivation is ASD-specific relative to the ADHD. This finding was significant in whole-brain analyses of ToM > RD contrast, and either significant or at trend-level in ROI analyses of both mentalizing contrasts. Previous studies have shown reduced rANG activation in children and adults with ASD relative to controls (15, 17–19). However, some studies have also found overactivation in the social brain regions during mentalizing in children and adults with ASD relative to TD (21–24), typically concomitant with unimpaired mentalizing task performance. The present findings could reflect increased processing effort in ASD to perform mentalizing as well as the TD controls and ADHD group [e.g., (23, 24)]. Supporting this interpretation was the positive correlation between rANG activation and the increased prompts for describing ToM movement in ASD, and between rANG activation and RT for classifying ToM clips in TD controls.

Discrepancies of findings were observed across contrasts (ToM > GD *vs*. ToM > RD) and analysis types (e.g., whole brain *vs*. ROI). Specifically, the ToM > GD contrast, relative to ToM > RD in whole-brain and ROI analyses, reduced some brain activation differences to non-significance. Thus, GD motion seems to evoke temporoparietal activation at intermediate level between RD and ToM, which could express parametric modulation of right temporoparietal activation when observing interaction based on goal pursuit alone compared to interaction involving the attribution of mental states in others.

Overall, the clinical groups showed a lack of increased FC during ToM compared to TD controls. The underconnectivity between rANG and bilateral TPJ/STS in particular seemed in line with the findings of anterior and posterior TPJ dysconnectivity between ASD and ADHD during resting state (79). The right-lateralized local rANG-rTPJ/STS underconnectivity during ToM is interesting, given the strong evidence of a positive association between BOLD activation and local FC density during mentalizing (80). Specifically, the rANG activation in both ASD groups, with and without ADHD, exhibited opposite effect to what is expected from a *reduced* local temporoparietal connectivity between rANG and rTPJ. This supports the atypical brain hypothesis [e.g., (21–24, 27)], instead of the hypo-intentionality hypothesis of ASD (20), which now extends to the comorbid group.

The lack of increased connectivity in rIFG-PCC during ToM, was found in ASD and ASD+ADHD but not in ADHD or TD controls. As well as implicated during mentalizing, the rIFG is part of the mirroring networks and is implicated in action processing and social attention. The rIFG is hypothesized as a gateway into the mentalizing networks (81, 82), which include the PCC. Thus, the rIFG-PCC underconnectivity may reflect information transmission failures across the two networks (26, 30), which is ASD-differentiating.

Relative to TD controls, ADHD and ASD+ADHD showed decreasing FC between mPFC and lTPJ with increasing ToM. Functional coupling between mPFC and the temporal cortices occurs bilaterally during mentalizing [e.g., (28, 75, 82, 83)]. While it has not been discussed as much as the rTPJ in the context of mentalizing, the lTPJ is implicated in explicit ToM in meta-analytic connectivity modeling (84), and lesions in the region impaired spontaneous ToM in adults (85, 86). Those specific subdomains of ToM difficulties could perhaps be investigated further in ADHD.

The findings of the study should be viewed with its strengths and limitations. Firstly, the sample size was relatively small, which may have reduced statistical power to detect small effects and increased probability of false positives. The inclusion of young adult males only enhanced the group homogeneity at the expense of the finding's generalizability to other population groups, including female, children, and older adults with the conditions. The absence of correlations between primary ToM measures and brain activation and connectivity also constrained the interpretation of findings. There was limited variance of task performance across all participant groups, which could have reflected reduced symptom severity in non-clinically referred participants (87) or a lack of sensitivity of the simplistic animated-triangle fMRI task for detecting differences between groups. Finally, it is also possible that ToM impairments are present in ADHD in a subtle form that was undetectable by the animated triangle task. Future studies could investigate clinically referred participants, using more complex and naturalistic mentalizing tasks [e.g., (18, 26)].

To summarize, despite evidence of reduced connectivity in all three clinical groups relative to TD controls during ToM, a differentially increased activation pattern was found in both ASD and comorbid groups relative to ADHD in right temporoparietal cortex, which is a key mentalizing region. Both ASD and comorbid groups also showed reduced right inferior frontal and posterior cingulate coupling, which may reflect an atypical information transmission to the mentalizing network. In contrast, both ADHD and comorbid groups showed decreasing connectivity between medial prefrontal and left temporoparietal cortices with increasing ToM when compared to TD controls. These findings denote a complex pattern of atypical brain function underpinning mentalizing in these three conditions in young adult males, with some evidence of ASD- and ADHD-differentiating features and a combined neurofunctional atypicality in the comorbid group.

## Data Availability Statement

The datasets presented in this study can be found in online repositories. The names of the repository/repositories and accession number(s) can be found at: https://osf.io/nrj8g/.

## Ethics Statement

The studies involving human participants were reviewed and approved by National Health Service Research Ethics Committee (NHS REC 13/LO/0373). The patients/participants provided their written informed consent to participate in this study.

## Author Contributions

SL, KR, and ES conceptualized the study. CMM, KA, and VS contributed to recruitment. SL conducted the recruitment and data collection. SL, DI, OO'D, and CM contributed to the analysis of the data. SL and DI drafted the manuscript. DL, CMM, CM, KA, VS, KR, and ES contributed to the manuscript preparation. All authors contributed to the article and approved the submitted version.

## Conflict of Interest

DI has received honoraria and travel support from Otsuka-Lundbeck and Janssen. KR has received a grant from Shire/Takeda for another study. The remaining authors declare that the research was conducted in the absence of any commercial or financial relationships that could be construed as a potential conflict of interest.

## Abbreviations

ADHD: attention-deficit/hyperactivity disorder
ASD: autism spectrum disorder
FC: functional connectivity
GD: goal directed movement
fMRI: functional Magnetic Resonance Imaging
TD: typically developing controls
mPFC: medial prefrontal cortex
ToM: Theory of mind
TPJ: temporoparietal junction
rANG: right angular gyrus
RD: random movement
ROI: region-of-interest.
