# Supplementary material for: Neural Correlates of Theory of Mind in Autism Spectrum Disorder, Attention-Deficit/Hyperactivity Disorder, and the Comorbid Condition
Source: Front Psychiatry. 2020 Nov 6;11:544482. doi: 10.3389/fpsyt.2020.544482 (PMC7677232; doi:10.3389/fpsyt.2020.544482)
Supplement: Supplementary file 1 [file Data_Sheet_1.PDF]

Supplementary Material

Figure S1: Schematic Diagram of Frith-Happé Animated Triangle Task

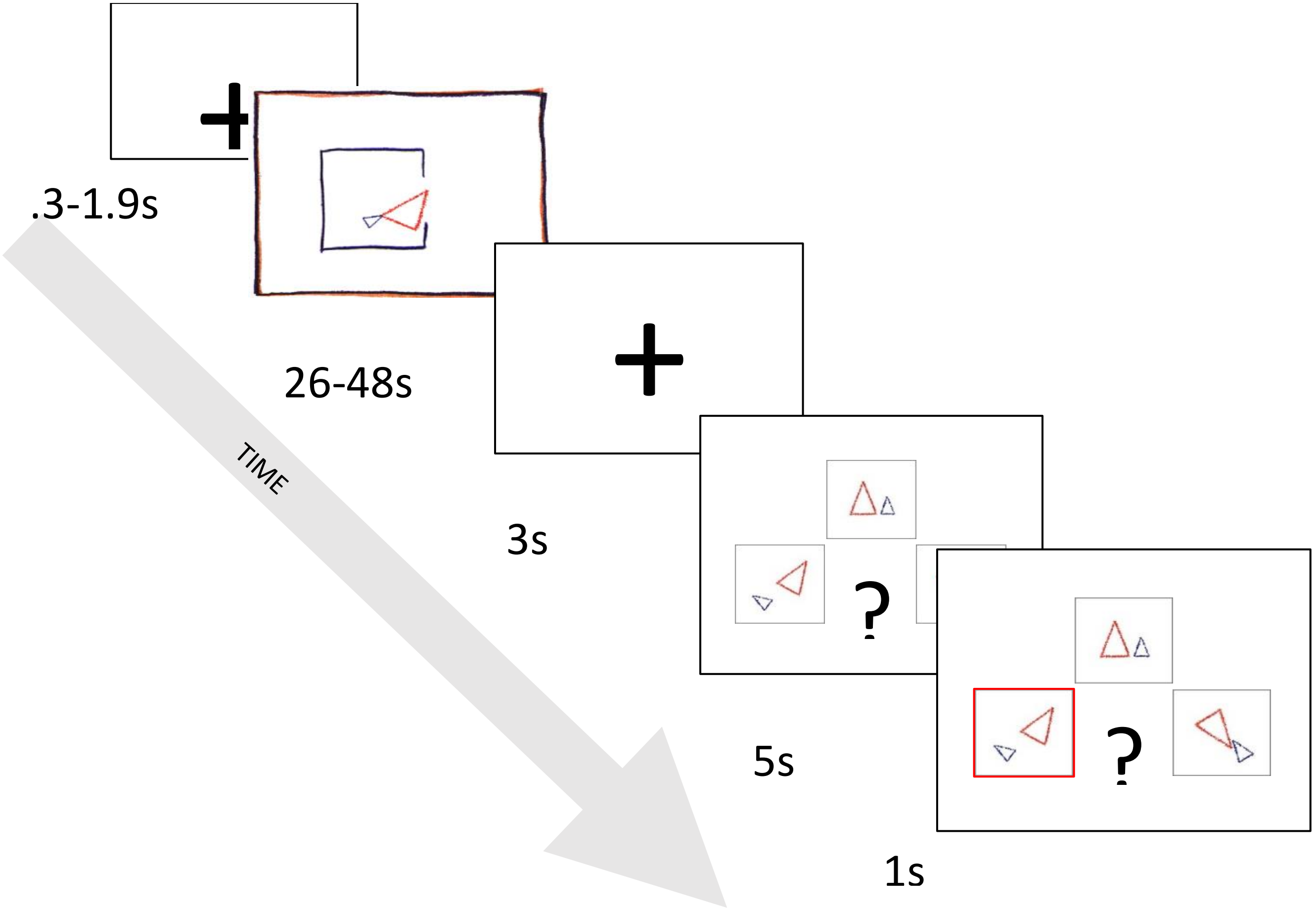

The task consists of twelve 26-48s cartoons depicting two triangles whose movement reflect (1) ToM or mentalizing, e.g. persuading, (2) Goal-directed (GD) or physical interaction, e.g. following and (3) Random (RD) purposeless motions, e.g. floating. Each movement condition is represented by four clips, shown in a pseudo-randomised order consistent across participants. A fixation cross of 1-s mean duration (range: 0.3 – 1.9 s) preceded each clip. The clip is then followed by 3-s fixation, followed by a visual multi-choice question asking the participants to identify the movement featured in the clip, i.e. ToM, GD, or RD, that had to be answered within a 5-s interval. The participant's choice is highlighted by a red box around it for a 1-s interval.

Table S1: Comparisons of Task Performance Across Groups

	Condition	TD (n = 25)	ASD (n = 19)	ASD+ADHD (n = 18)	ADHD (n =21)	Group effect	Condition effect	Condition x Group	*Post-hoc*
						F (*p*-value)			
Outside fMRI Task									
Intentionality	ToM	3.9 (3.6-4.1)	3.7 (3.2-4.1)	4.0 (3.7-4.3)[a]	3.8 (3.6-4.1)[b]	2.1 (.108)	239.0 (< .0001)	.68 (.57)	**TD**: ToM*** > GD<br>**ASD**: ToM*** > GD<br>**ASD+ADHD**: ToM*** > GD<br>**ADHD**: ToM*** > GD
	GD	2.5 (2.3-2.7)	2.3 (2.1-2.6)	2.7 (2.4-3.0)[a]	2.8 (2.5-3.0)[b]				
Appropriate-ness	ToM	1.3 (1.1-1.5)	1.1 (.92-1.4)	1.4 (1.2-1.6)[a]	1.3 (1.0-1.5)[b]	.88 (.455)	33.3 (< .0001)	1.6 (.19)	**TD**: GD*** > ToM<br>**ASD**: GD*** > ToM<br>**ADHD**: GD*** > ToM
	GD	1.7 (1.5-1.8)	1.5 (1.3-1.7)	1.5 (1.3-1.6)[a]	1.6 (1.5-1.8)[b]				
Length	ToM	11.6 (10.4-12.7)	8.5 (7.2-9.7)	10.4 (8.2-12.7)[a]	11.1 (9.8-12.4)[b]	2.7 (< .05)	117.3 (< .0001)	2.7 (.051)	**TD**: ToM*** > GD<br>**ASD**: ToM*** > GD<br>**ASD+ADHD**: ToM*** > GD<br>**ADHD**: ToM*** > GD<br>**/ToM**: TD***, ASD+ADHD**, ADHD*** > ASD<br>**/GD**: TD*, ASD+ADHD**, ADHD** > ASD
	GD	7.7 (6.4-9.0)	6.4 (5.4-7.4)	8.1 (6.2-10.1)[a]	8.1 (6.8-9.4)[b]				
Prompts	ToM	.45 (.20-.71)	1.1 (.54-1.6)	.79 (.31-1.3)[a]	.28 (.07-.48)[b]	3.5 (.02)	184.3 (< .0001)	1.83 (.15)	**TD**: GD *** > ToM<br>**ASD**: GD *** > ToM<br>**ASD+ADHD**: GD *** > ToM<br>**ADHD**: GD *** > ToM<br>**/ToM**: TD*, ADHD*** > ASD
	GD	2.4 (1.7-3.2)	2.4 (2.0-2.8)	2.4 (1.8-2.9)[a]	2.1 (1.7-2.5)[b]				
Inside fMRI Task									
Correct answer during fMRI (%)	ToM	96 (92-100)	95 (88-100)	88 (79-96)	94 (89-100)	2.5 (.07)	42.3 (< .0001)	1.64 (.14)	**TD**: ToM, RD*** > GD<br>**ASD**: ToM, RD*** > GD<br>**ASD+ADHD**: ToM, RD*** > GD<br>**/GD**: ADHD** > ASD, ASD+ADHD
	GD	76 (68-84)	69 (60-79)	68 (55-81)	85 (77-92)				
	RD	92 (86-98)	95 (88-100)	89 (81-97)	92 (87-98)				
Reaction Time (s)	ToM	.91 (.76-1.06)	1.03 (.79-1.26)	1.26 (.95-1.57)	1.00 (.78-1.23)	2.6 (.06)	.74 (.48)	.88 (.52)	**/ToM**: TD* < ASD+ADHD<br>**/GD**: TD* < ASD, ADHD<br>**/RD**: TD* < ASD+ADHD
	GD	.85 (.70-1.01)	1.12 (.90-1.35)	1.08 (.86-1.30)	1.10 (.88-1.32)				
	RD	.86 (.76-.97)	1.07 (.86-1.27)	1.23 (.92-1.54)	1.14 (.91-1.36)				

Abbreviations: TD = typical development, ASD = autism spectrum disorder, ADHD = attention-deficit/hyperactivity disorder, ToM = theory of mind condition, GD = goal-directed condition, RD = random condition. [a]: n=17; [b]: n=20. Post-hoc significant threshold: **p* < .05, ***p* < .01, ****p* < .001, with Tukey-Kramer multiple comparison correction.
